# Effects of continuous and intermittent renal replacement therapies among adult patients with acute kidney injury

**DOI:** 10.3205/hta000127

**Published:** 2017-03-01

**Authors:** Tonio Schoenfelder, Xiaoyu Chen, Hans-Holger Bleß

**Affiliations:** 1 IGES Institut GmbH, Berlin, Germany

**Keywords:** acute kidney injury, acute renal failure, hemodialysis, hemodiafiltration, intensive care unit, renal dialysis, renal replacement therapy, intermittent renal replacement therapy, continuous renal replacement therapy, health technology assessment, HTA, meta-analysis, IRRT, CRRT, SLED, renal recovery

## Abstract

**Background:** Dialysis-dependent acute kidney injury (AKI) can be treated using continuous (CRRT) or intermittent renal replacement therapies (IRRT). Although some studies suggest that CRRT may have advantages over IRRT, study findings are inconsistent. This study assessed differences between CRRT and IRRT regarding important clinical outcomes (such as mortality and renal recovery) and cost-effectiveness. Additionally, ethical aspects that are linked to renal replacement therapies in the intensive care setting are considered.

**Methods:** Systematic searches in MEDLINE, EMBASE, and Cochrane Library including RCTs, observational studies, and cost-effectiveness studies were performed. Results were pooled using a random effects-model.

**Results:** Forty-nine studies were included. Findings show a higher rate of renal recovery among survivors who initially received CRRT as compared with IRRT. This advantage applies to the analysis of all studies with different observation periods (Relative Risk (RR) 1.10; 95% Confidence Interval (CI) [1.05, 1.16]) and to a selection of studies with observation periods of 90 days (RR 1.07; 95% CI [1.04, 1.09]). Regarding observation periods beyond there are no differences when only two identified studies were analyzed. Patients initially receiving CRRT have higher mortality as compared to IRRT (RR 1.17; 95% CI [1.06, 1.28]). This difference is attributable to observational studies and may have been caused by allocation bias since seriously ill patients more often initially receive CRRT instead of IRRT. CRRT do not significantly differ from IRRT with respect to change of mean arterial pressure, hypotensive episodes, hemodynamic instability, and length of stay. Data on cost-effectiveness is inconsistent. Recent analyzes indicate that initial CRRT is cost-effective compared to initial IRRT due to a reduction of the rate of long-term dialysis dependence. As regards a short time horizon, this cost benefit has not been shown.

**Conclusion:** Findings of the conducted assessment show that initial CRRT is associated with higher rates of renal recovery. Potential long-term effects on clinical outcomes for more than three months could not be analyzed and should be investigated in further studies. Economical analyzes indicate that initial CRRT is cost-effective when costs of long-term dialysis dependence are considered. However, transferability of the economic analyzes to the German health care system is limited and the conduction of economical analyzes using national cost data should be considered.

## Background

Acute kidney injury (AKI) is a common complication in critically ill patients and associated with increased in-hospital mortality and risk of chronic dialysis as well as with high treatment costs [1], [2], [3]. The incidence of AKI in the intensive care unit (ICU) ranges from approximately 20% to 50%, depending on the population studied [4]. In severe AKI, a renal replacement therapy (RRT) might be required. RRT is typically provided intermittently (IRRT) or continuously (CRRT). The modalities used differ according to the mechanism they remove fluids and toxins.

IRRT describe various blood purification techniques that are not applied continuously. Intermittent hemodialysis (IHD) is administered at variable intervals, typically for 3 to 6 hours per treatment. Advantages of IHD are prompt therapeutic effects (e.g., in case of life-threatening hyperkalemia) due to fast removal of toxins and down-times due to the restricted treatment period, which allows for diagnostic interventions, operations, and mobilization of patients [5], [6]. The most common complication of IHD is hypotension, which affects approximately 20% to 30% of all treatments. Some of the causes are dialysis specific, such as the rapid volume removal and changes of plasma osmolality [7]. Particularly in critically ill, hemodynamically unstable patients, this complication may lead to further organ ischemia and injury [7]. Sustained low efficiency dialysis (SLED) or slow extended daily dialysis (SLEDD) and extended daily hemofiltration (EDHF) are characterized by a prolonged duration of dialysis between 6 to 12 hours per treatment. Compared to IHD, in these methods solute clearance is slower, blood flow is lowered, and fluid removal is more gradual [5], [7], [8].

CRRT are intended to run for 24 hours per day. In comparison to IRRT, the total amount of solute transported per unit of time is less. However, since administered over a period of 24 hours, total solute clearance may exceed that achieved with IRRT [6], [7]. Since fluids are removed more slowly, CRRT may result in better hemodynamic stability and better control of fluid balance [6]. Other advantages of CRRT are the improved efficiency of solute removal and the capacity to adapt the treatment to the patient’s need at any time [6], [7]. Disadvantages of CRRT are the need for immobilization, the use of continuous anticoagulation, and its costs, which are significantly higher as compared with IHD [6], [9].

Despite the theoretical advantages of CRRT over IRRT, study findings on the effect of RRT modalities on outcomes of AKI patients are inconsistent. However, most currently available systematic reviews, meta-analyzes, and health technology assessments [10], [11], [12] are based on studies that were published several years ago. They may not reflect the present state of research and, therefore, may not consider technical advancements adequately. Additionally, these studies primarily rely on randomized controlled trials (RCTs), which often enroll selected patients that may significantly differ from the typical AKI patient, and often consist of small study samples, which may limit generalizability of study results [6], [13], [14], [15]. In contrast, large observational studies, including unselected patient populations receiving RRT, indicate significant differences between CRRT and IRRT concerning relevant patient outcomes such as renal recovery [16], [17], which were confirmed by a recently conducted systematic review [18]. 

Since study findings on the effect of choice of RRT modality on patients’ outcomes are inconsistent, a comprehensive assessment, including RCTs as well observational studies, is necessary. Accordingly, this assessment aimed to systematically review the current literature and to analyze data on safety, efficacy, economic issues, as well as on ethical and social aspects among patients with AKI requiring RRT.

## Research questions

The present assessment was guided by the following research questions:

### Medical research questions

Are there differences between CRRT and IRRT regarding:

- mortality,
- renal recovery among survivors,
- hemodynamic tolerance,
- fluid balance,
- length of stay (LOS) in ICU, LOS in hospital,
- and health related quality of life?

### Health-economic research question

Comparing IRRT to CRRT, are there any differences in the cost-effectiveness?

### Ethical and social research questions

Which ethical aspects should be considered in decisions on initiating RRT?

Are there any criteria influencing the decision on prescribing RRT?

## Methods

### Study selection criteria

The conducted assessment included critically ill patients who received RRT for AKI.

As regards interventions, the term CRRT was used to describe continuous venovenous hemofiltration (CVVHF), continuous venovenous hemodialysis (CVVHD), continuous venovenous hemodiafiltation (CVVHDF), and slow continuous ultrafiltration (SCUF). Continuous arteriovenous RRT were not included in this assessment as such therapies are nowadays rarely applied, have largely been replaced by CRRT, and are only used in emergency cases if venovenuous therapies are not available [7], [19], [20]. IRRT was used to describe IHD, SLED, SLEDD, EDHF, and prolonged intermittent renal replacement therapy (PIRRT).

For medical outcomes, RCTs and observational studies comparing CRRT and IRRT were included. Concerning ethical and social issues, also studies with lower levels of evidence such as expert opinions and narrative reviews were considered. To assess economical aspects, studies presenting cost-effectiveness analyzes were factored in. Only full-text versions of publications in German or English language were included.

### Search process for study identification

A systematic literature search was performed which comprised of electronic database searches in MEDLINE, EMBASE, and the Cochrane Library. Additionally, manual searches on pertinent websites were conducted and the bibliography of identified literature was reviewed. Studies were included from 1995 onwards. Searches were carried out in December 2014. Titles and abstracts of identified references were screened according to predefined selection criteria. Subsequently, full-text versions of selected publications were examined. 

### Assessment of study methodology and data extraction

The methodological quality of the studies was assessed by means of fixed criteria. As regards observational studies and RCTs, the Downs and Black Scale was used [21]. Economic studies were assessed using the Checklist by Drummond and Jefferson [22]. Study selection, methodological assessments, and data extraction were done by two reviewers independently.

### Synthesis of results

Extracted data of studies addressing medical research questions were pooled using a random effects-model. For dichotomous outcomes, relative risk (RR) with 95% confidence interval (CI) were used to pool results, and for metric data Hedges’ g was used. For patients who received CRRT as well as IRRT (crossover), outcomes were compared according to the initial RRT modality applied on an intention-to-treat (ITT) basis. Pooled analysis were stratified according to study design (e.g., RCTs vs. observational studies). Statistical heterogeneity for pooled results was quantified using Cochran’s Q and the I² statistics [23]. All statistical calculations were performed using SAS 9.3. Regarding study data that were not suitable for meta-analyzes (e.g., study information on ethical and social aspects), results were summarized and presented in text format.

## Results

### Study selection

The systematic literature search yielded 5,823 references. Additionally, 24 studies were identified by manual search. Exclusion of double publications left 4,408 citations. Of these, 138 publications were considered potentially relevant and acquired in full-text. Finally, 49 studies met the inclusion criteria and were included in this assessment. Of those, 42 studies presented outcome data for mortality and morbidity, 3 presented data for cost-effectiveness, and 4 information on ethical and social aspects (see Attachment 1).

**Figure 1:**
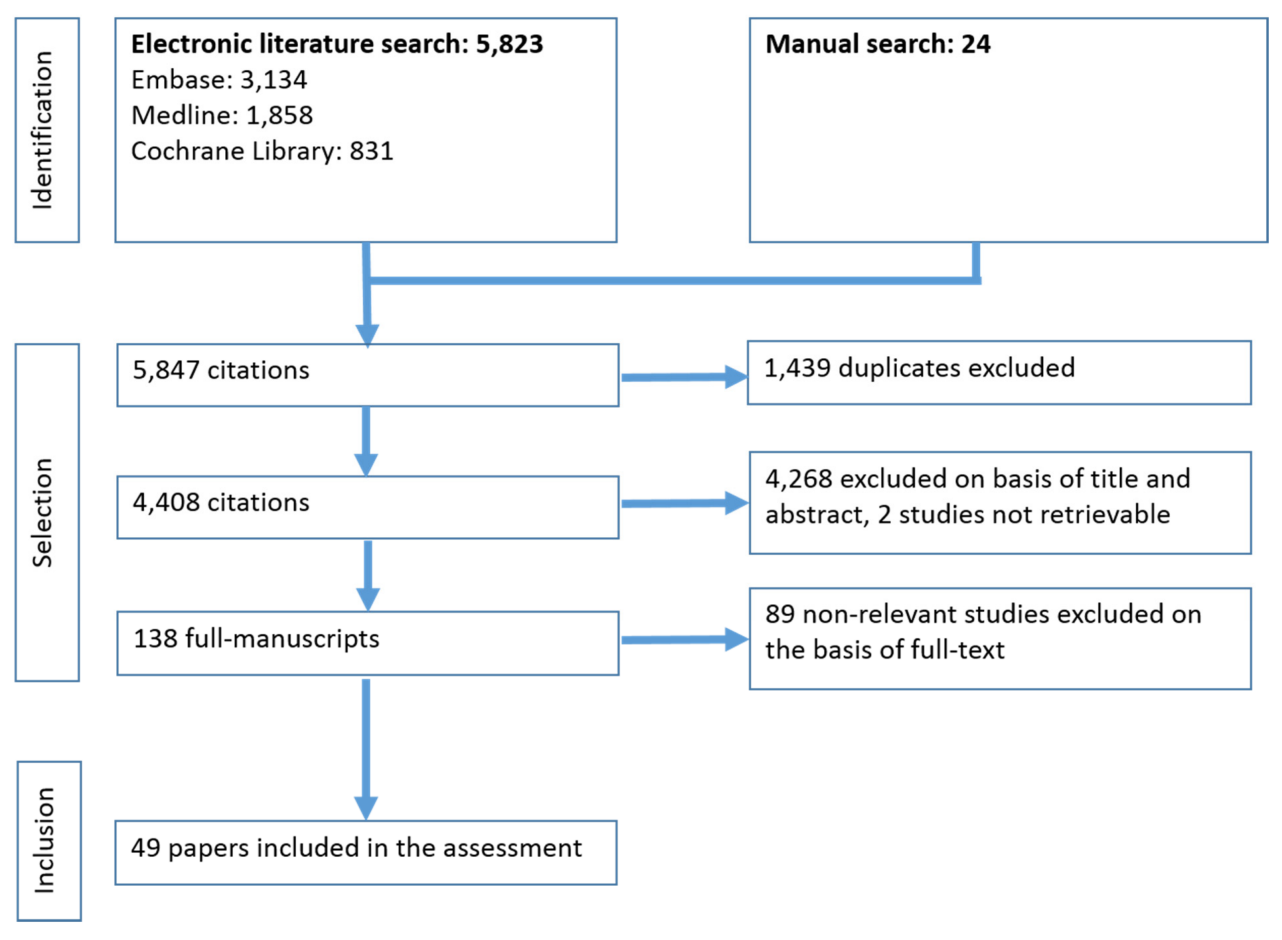
Study selection process

The study selection process is presented in Figure 1 (Fig. 1).

### Medical evaluation

Conducted analyzes show significant differences between CRRT and IRRT regarding some of the outcomes assessed.

#### Mortality

**Figure 2:**
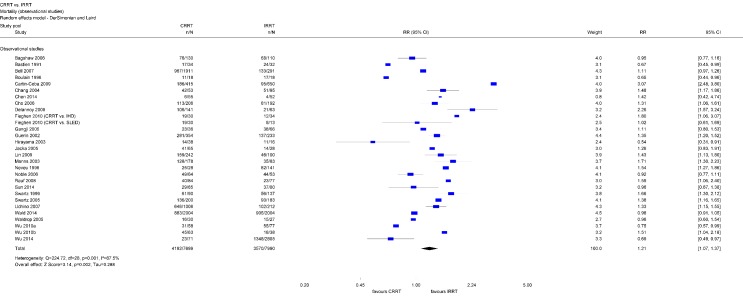
Forest plot for mortality (observational studies)

Patients initially receiving CRRT have higher mortality as patients initially receiving IRRT (Relative Risk (RR) 1.17; 95% Confidence Interval (CI) [1.06, 1.28]). This difference primarily is attributable to observational studies (RR 1.21; 95 % CI [1.07, 1.37]) (Figure 2 (Fig. 2)) and most likely is due to allocation bias, since seriously ill patients more often initially receive CRRT instead of IRRT [6], [24], [25]. 

**Figure 3:**
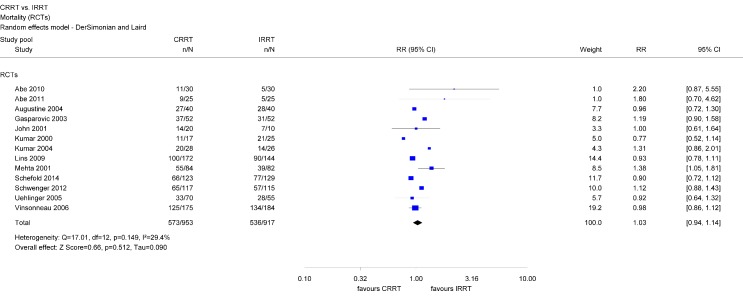
Forest plot for mortality (RCTs)

Within RCTs, there was no statistically significant difference regarding mortality between both modalities (RR 1.03; 95 % CI [0.94, 1.14]) (Figure 3 (Fig. 3)). Separate analyzes of 30-day, 60-day, and 90-day mortality did also show no statistically significant difference between CRRT and IRRT (Table 1 (Tab. 1)).

There was evidence of substantial heterogeneity across observational studies and of moderate heterogeneity across RCTs concerning the analysis of 30-day-mortality (Table 1 (Tab. 1)).

#### Renal recovery among AKI survivors

**Figure 4:**
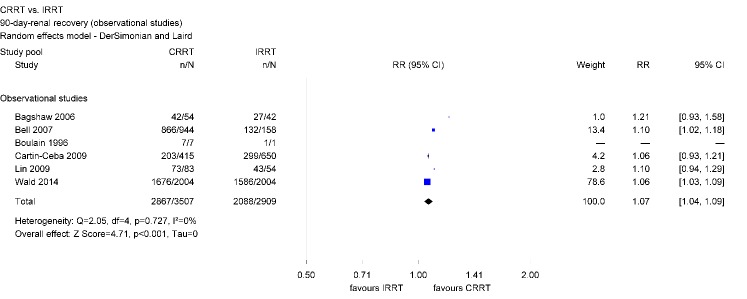
Forest plot for 90-day-renal recovery among survivors

Pooled analyzes of all 26 identified studies show a higher rate of renal recovery among AKI survivors who initially received CRRT as compared with IRRT. About 82% of survivors in the CRRT group and 71% of survivors in the IRRT group had renal recovery. This advantage applies to the analysis of all identified studies with different observation periods (RR 1.10; 95% CI [1.05, 1.16]) as well as to a selection of studies which reported an observation period of 90 days after initial treatment (RR 1.07; 95% CI [1.04, 1.09]) (Figure 4 (Fig. 4)). 

As regards observation periods longer than three months, there are no significant differences between RRT. However, only two studies have been identified and analyzed (RR 0.97; 95% CI [0.43, 2.18]). 

**Figure 5:**
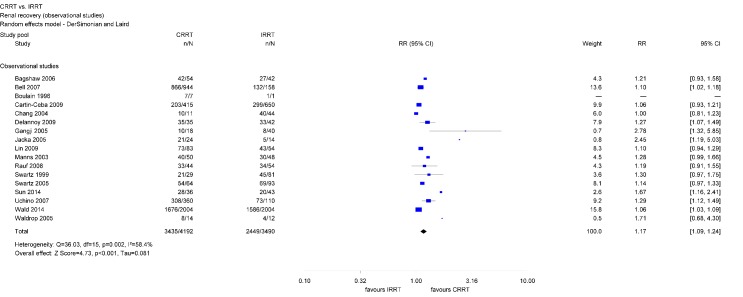
Forest plot for renal recovery among survivors (observational studies)

Differences between CRRT and IRRT regarding renal recovery primarily rely on observational studies. In total, 17 observational studies were analyzed. Survivors, initially treated with CRRT had a 17% higher chance of renal recovery than survivors treated initially with IRRT (RR 1.17; 95% CI [1.09, 1.24]) (Figure 5 (Fig. 5)).

**Figure 6:**
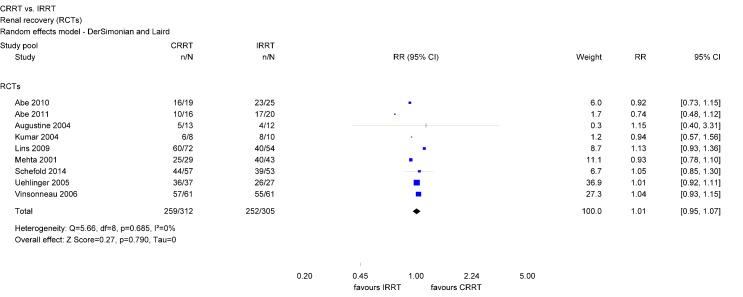
Forest plot for renal recovery among survivors (RCTs)

Separate analyzes of RCTs only did not show a statistically significant effect of dialysis modality (RR 1.01; 95% CI [0.95, 1.07]) (Figure 6 (Fig. 6)).

There was evidence of moderate heterogeneity concerning the analyzes including all observational studies and of substantial heterogeneity across the two studies with observation periods longer than 90 days. However, there was no heterogeneity across observational studies investigating 90-day-renal recovery (Table 2 (Tab. 2)).

#### Hemodynamic tolerance

This end-point analyzed study data regarding change in mean arterial pressure (MAP), hypotension, and hemodynamic instability. Therefore, study data of this end-point has been analyzed separately.

##### MAP

As regards MAP, one study has been identified [26]. This study included 40 patients receiving CRRT and IRRT, respectively. Findings show that CRRT do not significantly differ from IRRT with respect to change of MAP during dialysis (Hedges’ g=-0.45; 95% CI [–0.89, 0.00].

##### Hypotension

The literature search identified three RCTs and one observational study. Choice of renal replacement modality is not significantly associated with hypotension during dialysis (RR 0.71; 95% CI [0.39, 1.31]) when all identified studies were analyzed. There was evidence of substantial heterogeneity (Table 3 (Tab. 3)).

**Figure 7:**
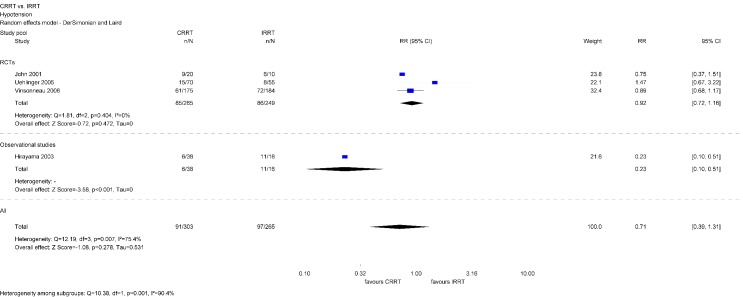
Forest plot for hypotension

However, depending on study design, findings are inconsistent. While the identified observational study shows that hypotension occurred less frequently among patients treated with CRRT, separate analyses of RCTs did not find an effect (Figure 7 (Fig. 7)).

##### Hemodynamic instability

**Figure 8:**
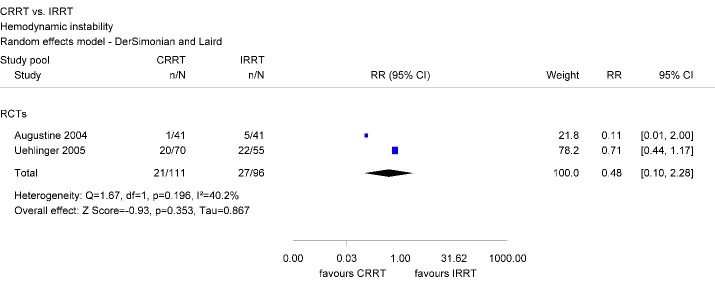
Forest plot for hemodynamic instability

In total, two studies have been identified. Hemodynamic instability occurred in approximately 19% in the CRRT group and 28% in the IRRT group (Figure 8 (Fig. 8)).

Differences were statistically non-significant (RR 0.48; 95% CI [0.10, 2.28]). There was no evidence of significant heterogeneity across both studies (Table 4 (Tab. 4)).

#### Fluid balance

Data on fluid balance was presented by four identified RCTs. Due to differing definitions of that endpoint, data was not pooled. Three RCTs did not report statistically significant differences in fluid balance between dialysis modalities [26], [27], [28]. One study, which randomized 40 patients to CRRT and IRRT, respectively, reported a greater net volume removal in the CRRT group during 72 hours of dialytic treatment [29].

#### Length of stay

**Figure 9:**
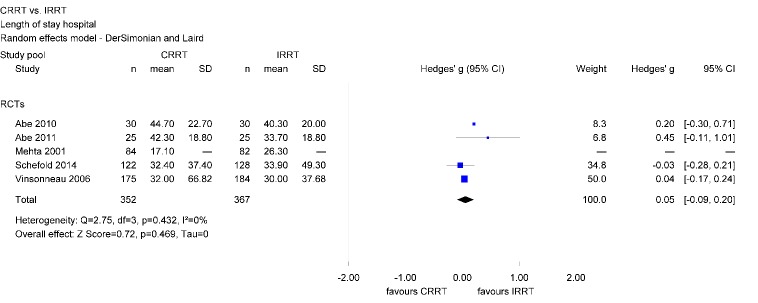
Forest plot for length of stay in hospital

Within the literature search, in total 11 comparisons of CRRT and IRRT concerning LOS in hospital as well as for LOS in ICU have been identified. The analyzes show no statistically significant effects of dialysis modality on number of days spent in hospital (Hedges’ g=0.05; 95% CI [–0.09, 0.20]) (Figure 9 (Fig. 9)).

**Figure 10:**
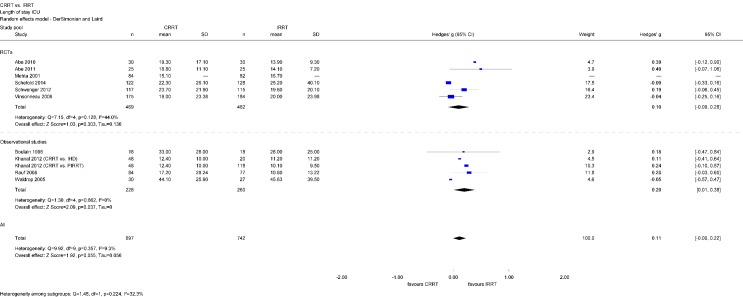
Forest plot for length of stay in ICU

There was also no difference between dialysis modalities in LOS in ICU (Hedges’ g=0.11; 95% CI [–0.00, 0.22]). A separate analysis of the identified observational studies revealed a statistically significant reduction of LOS in ICU among patients treated with IRRT (Hedges’ g=0.20; 95% CI [0.01, 0.38]), however, the effect size was very small. A separate analysis of RCTs only did not show any significant differences regarding LOS in ICU (Figure 10 (Fig. 10)).

There was no evidence of significant heterogeneity across studies (Table 5 (Tab. 5)).

#### Health-related quality of life 

No studies were identified which compared health-related quality of life among AKI patients who either received CRRT or IRRT.

### Economical evaluation

In total, three cost-effectiveness studies were identified which compared CRRT and IRRT. Studies were conducted from payors’ perspective in USA [30], Belgium [31], and Canada [32]. Characteristics of the studies are presented in Table 6 (Tab. 6). Data on cost-effectiveness of CRRT and IRRT is inconsistent. Recent analyzes indicate that initial CRRT among AKI patients is cost-effective compared to initial IRRT due to a reduction of the rate of long-term dialysis dependence. This applies to periods of five years (incremental cost effectiveness ratio (ICER), undiscounted: –116,121 US$; ICER: –106,527 US$, discounted) and a lifelong time horizon (ICER: –196,956 US$). In this analysis, the five-year total cost on average was lower for CRRT patients (37,780 US$) than for IRRT patients (39,448 US$) [30]. However, an analysis relying on older study data contradicts these findings and shows that IRRT is less cost intensive than CRRT on a lifelong time horizon (96,635 C$ vs. 100,314 C$; ICER not shown in study) [32]. As regards short time periods, the cost benefit of CRRT has not been shown. A one-year analysis resulted in an ICER of 400,701 US$ [30] and a two-year analysis in an ICER of 114,012 € [31] (both discounted).

In conducting the assessment, no studies were identified, which based on data of the German health care system. The transferability of the referenced economic analyzes may be limited due to varying health care structures. 

## Ethical and social aspects

### Ethical aspects to be considered in decisions on initiating RRT

Since data presented did not allow quantitative analyzes, a summary of study findings is being presented. In total, two studies were identified that addressed ethical aspects of RRT among AKI patients in the ICU [33], [34]. Although technically feasible in the majority of patients, in particular due to the availability of CRRT, dialysis may not benefit all AKI patients in the ICU. With regard to that matter, authors discuss high mortality rates among those patients and the importance to consider patients’ prognosis when making decisions to initiate dialysis. Concerning dialysis withholding and withdrawing, authors stated the importance of autonomy as regards decision making; the patients or their proxy must consent. Study data show, that when dialysis was initiated and continued, the decision-making process included patient or family members in every other case. When dialysis was withdrawn, discussion with the patient or family members were documented in 83% of cases and when dialysis was withheld, discussion with the patient or family members was documented in 63% of cases [34]. The ethical principle of beneficence also plays an important role; the benefits must outweigh the risks. The patient’s prognosis addresses the ethical principle of beneficence. In patients with terminal illness or if patient’s medical condition precludes the technical process of dialysis, withholding and withdrawing dialysis may be considered.

### Criteria influencing the decision on prescribing RRT

Two studies presented data regarding choice of renal replacement therapy. Data is based on one survey including members of the European Society of Intensive Care Medicine (ESICM) (N=272) [35] and on one survey that included 387 ICUs form 349 German hospitals [36]. As regards prescription of RRT, in most cases nephrologists were not involved in decision-making process. The survey including members of the ESICM showed that decision on initiation of RRT was taken in 7.4% of cases by nephrologists. The survey including German ICUs revealed that nephrologists were involved in 22% of cases. Decisions on initiation of RRT were mostly taken by intensivists (92.6%) [35] and anaesthesists (53%) [36]. CRRT was available in most ICUs/hospitals whereas IRRT were less frequently available. Most of the prescribers preferred using CRRT. Reasons were perception of better hemodynamic stability, easier temperature control, better therapeutic effect, and easier fluid balance control.

## Discussion

### Key findings

In the conduction of this assessment, a systematic review has been performed, which identified 49 relevant studies reporting data on medical, economical, as well as on ethical and social aspects among AKI patients receiving RRT in the ICU. Study findings indicate significant effects of choice of RRT on some patient-relevant outcomes. 

Analyzes of mortality data indicate significant differences between patients treated using CRRT or IRRT. The pooled analysis using the entire study data shows a higher mortality rate among CRRT patients. However, this finding was not consistent across different types of studies since it did not reach statistical significance amongst RCTs and only relies on observational studies. Analyzes conducted by other authors agree on that finding [10], [11], [12], [37], [38]. For example, a meta-analysis including 17 RCTs did not find an impact of dialysis modality on mortality, however, when observational studies were factored in, mortality rate in patients treated with CRRT was significantly higher [37]. The difference in mortality rates according to dialysis modality might be caused by allocation bias, since CRRT are often preferred in critically ill, hemodynamically unstable patients [5], [6], [39]. However, these patients typically suffer from bad health in general and, consequently, tend to have higher mortality risk. Several observational studies show that patients who are treated using CRRT are more severely ill (e.g., Apache II), have higher numbers of organ failures, and require mechanical ventilation and vasopressor drugs more frequently than patients treated using IRRT [40], [41], [24], [42]. A recently published large observational study by Wald et al. confirms this argument [17]. This study used propensity matching considering more than 40 variables for treatment allocation to minimize the risk of allocation bias. Findings show that mortality rates rather depend on differences in patients’ characteristics than on dialysis modality.

Renal recovery has important implications for the patient and the health care system since it is associated with the patient’s quality of life and can result in cost savings by reducing the rate of long-term dialysis dependence [30], [43]. Findings regarding renal recovery show that AKI survivors initially receiving CRRT have a 10% higher chance of renal recovery as compared with those initially receiving IRRT. Separate analyzes of observational studies show even a 17% higher chance when initially treated with CRRT. Differences in the characteristics of CRRT and IRRT survivors due to the differing mortality rates may be present, but could not be assessed since studies did not present corresponding data. In contrast to observational studies, analyzed data of RCTs only did not reach statistical significance. However, RCTs consisted of relatively small study samples. The limited number of patients in RCTs may limit the precision of the estimates and the robustness of the findings. In contrast, within observational studies, the CRRT group consisted of approximately 4,200 survivors and the IRRT group of about 3,500. Those findings are consistent with other meta-analyzes. Schneider et al. included observational studies as well as RCTs published between 2000 and 2012 and reported that AKI survivors initially receiving IRRT have a 1.7 times higher risk of dialysis dependence than patients initially receiving CRRT [18]. Differences were largest within analyzed observational studies and statistically not significant within RCTs. Rabindranath et al. restricted their analyzes to RCTs and did not find a difference between RRT modalities regarding renal recovery [11]. 

The Kidney Disease: Improving Global Outcomes (KDIGO) foundation identified a research gap concerning the effect of RRT modalities on the long-term need for dialysis [6]. The analysis of studies which assessed renal recovery three months after initial treatment suggests that patients initially treated with CRRT have a lower risk of long-term dialysis dependence. However, as regards observation periods longer than three months, there were no significant differences between RRT modalities. This analysis based on only 2 studies comprising 234 survivors and, therefore, more studies are needed for reliable conclusions on long-term effects of choice of RRT.

In this assessment, a large number of studies with differing study methodology was analyzed. Depending on their design and source of data, multiple indicators for renal recovery were reported, which should be considered when interpreting study findings. Most studies defined renal recovery as independence from RRT without, however, presenting a clear definition for renal recovery or a set of parameters; in contrast a smaller part of the studies used defined parameters such as glomerular filtration rate and serum creatinine values. These differences in renal recovery definitions can be ascribed to the level of detail contained in the data analyzed in the studies: prospective trials provide different types of data than retrospective analyses basing on medical records or registers.

Better hemodynamic tolerance is often cited as an advantage of CRRT. Change in MAP, hypotension, and hemodynamic instability were investigated in the present assessment. Findings do not confirm this perception as analyzed study data do not show any significant difference between patients treated with CRRT or IRRT. 

As regards ICU length of stay, the pooled analyzes shows no significant difference between either group for having shorter LOS. Although a separate analysis of observational studies showed statistically significant differences favouring IRRT, the effect size was small and the confidence interval’s lower limit is close to 0. Concerning duration of hospital stay, there was no significant difference between CRRT and IRRT.

Data on cost-effectiveness is inconsistent. A recent analysis conducted by Ethgen et al. shows over a period of 5 years and a lifetime horizon that initial CRRT is cost-effective compared to initial IRRT due to a reduction of the rate of long-term dialysis dependence [30]. As regards short time horizons of 1 and 2 years, CRRT creates higher costs than IRRT [30], [31]. However, Klarenbach et al. contradict those findings and did not show any cost advantages of CRRT as compared with IRRT over a lifetime horizon [32]. In interpreting those inconsistent findings, differences in model specifications (such as time horizons, discounting, and reference year) as well as variations in the consideration of clinical end-points should be taken into account. These differences prevent a direct comparison between studies. For example, the analysis of Ethgen et al. based on data of a recently conducted, large observational study which used a robust propensity score to match over 2,000 CRRT and IRRT patient pairs [17]. Study results show a higher rate of dialysis dependence in the IRRT group [17] which transfers into higher long-term costs [30]. In contrast, Klarenbach et al. used data of an earlier review [10], which analyzed 5 RCTs comprising only 308 patients, which did not show differences in renal recovery between CRRT and IRRT [32]. De Smedt et al. did not include the outcomes dialysis dependence or renal recovery in their model [31] and, therefore, associated potential cost effects are not considered. 

The present assessment could not identify cost-effectiveness analyzes which based on data of the German health care system. All included studies analyzed national cost data (USA, Belgium, Canada), which are not related to the German health care context. The transferability of the referenced economic analyzes may be limited due to varying health care structures. Therefore, the conduction of cost-effectiveness analyzes on the effect of choice of RRT among AKI patients that give appropriate consideration to the German health care context is necessary.

### Study methodology

The present study systematically assessed the effect of choice of RRT on medical and economical outcomes amongst AKI patients requiring RRT and also addressed ethical issues. In total, 49 relevant studies were identified which included data of several thousand patients.

The strengths of this assessment are that it represents a comprehensive systematic review basing on a search strategy, which included major medical electronic databases. Study selection, assessment of methodological quality of selected studies, and data extraction were performed by two investigators independently. To our knowledge, this assessment is the most up-to-date analysis of published studies that have compared CRRT and IRRT in AKI patients and it also assessed many relevant outcomes in addition to mortality and renal recovery such as hemodynamic stability and LOS. Furthermore, the assessment includes both RCTs and observational studies to consider all relevant information. Most of the associations found depend on observational studies and, therefore, might be influenced by allocation bias. However, RCTs alone are generally not sufficient for the conduct of HTAs due to their limited sample size, length of follow-up, and inclusion of selected patient populations. It is suggested that information relevant for HTAs should also be obtained from non-experimental sources [44]. Most of the identified observational studies included large samples of unselected patients and might reflect treatment of AKI patients requiring RRT more realistically than RCTs, which often include highly selected patient populations [15].

As regards data analyzes, the ITT principle was used. However, in many studies patients crossed between modalities; in most cases patients initially receiving CRRT changed modality to IRRT than vice versa. Often such data are not reported by studies. Crossovers from one modality to the other may have influenced results of the present assessment. Some of the analyzes conducted indicate moderate or substantial heterogeneity across studies [23]. This primarily applies to mortality data of observational studies and should be considered when interpreting those results. However, observational studies did not provide sufficient data for the conduction of proper subgroup analyzes.

## Conclusion

Among survivors of AKI initial treatment with CRRT is associated with higher rates of renal recovery. Potential long-term effects on patient-relevant endpoints for more than three months could not be analyzed on the basis of currently available research and should be investigated in further studies. AKI patients initially receiving CRRT have higher mortality than patients initially receiving IRRT. Differences were primarily observed in observational studies and are likely caused by allocation bias since CRRT are often preferred in critically ill, hemodynamically unstable patients. This assessment did not find any relevant advantages of neither modality concerning change in MAP, hypotension, and hemodynamic instability as well as LOS in ICU and hospital. Economical analyzes indicate that initial CRRT is cost-effective as compared with initial IRRT when costs of long-term dialysis dependence are considered. However, transferability of the economic analyzes to the German health care system is limited and, therefore, conduction of economical analyzes using national cost data are necessary.

## Notes

### Competing interests

The conduction of this assessment and compilation of the report was supported by a funding of Baxter Deutschland GmbH. The authors had the entire formal and substantial control. 

## Supplementary Material

Included studies for assessed outcomes

## Figures and Tables

**Table 1:**
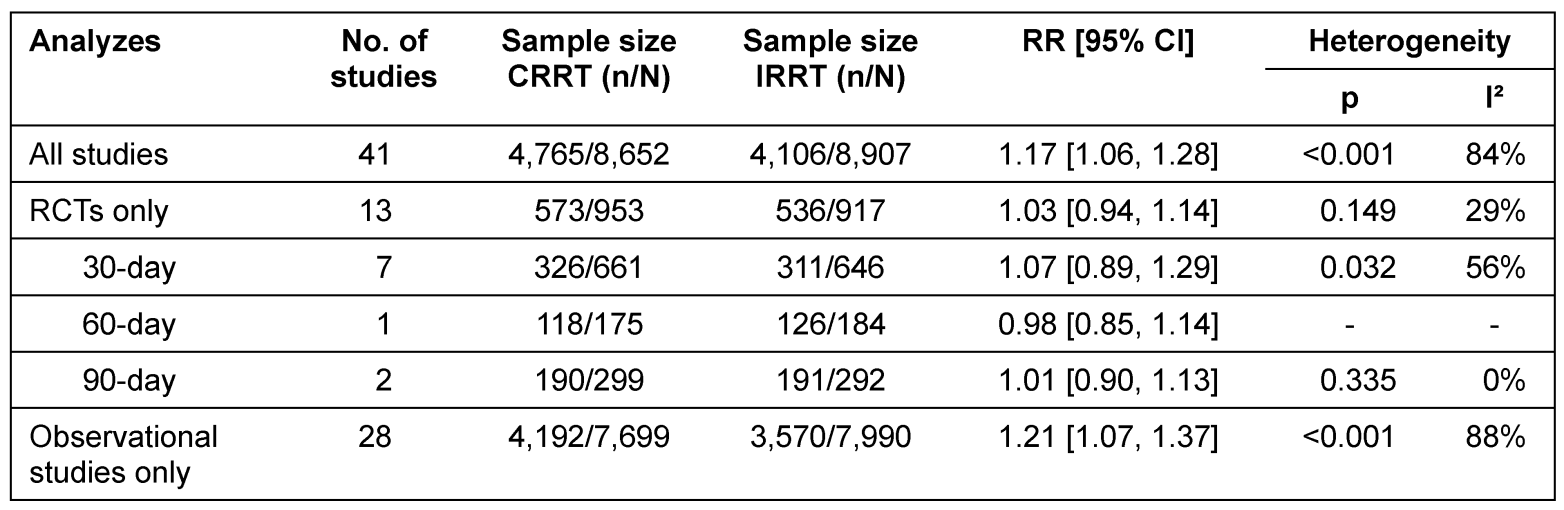
Effect-size summary statistics for mortality

**Table 2:**
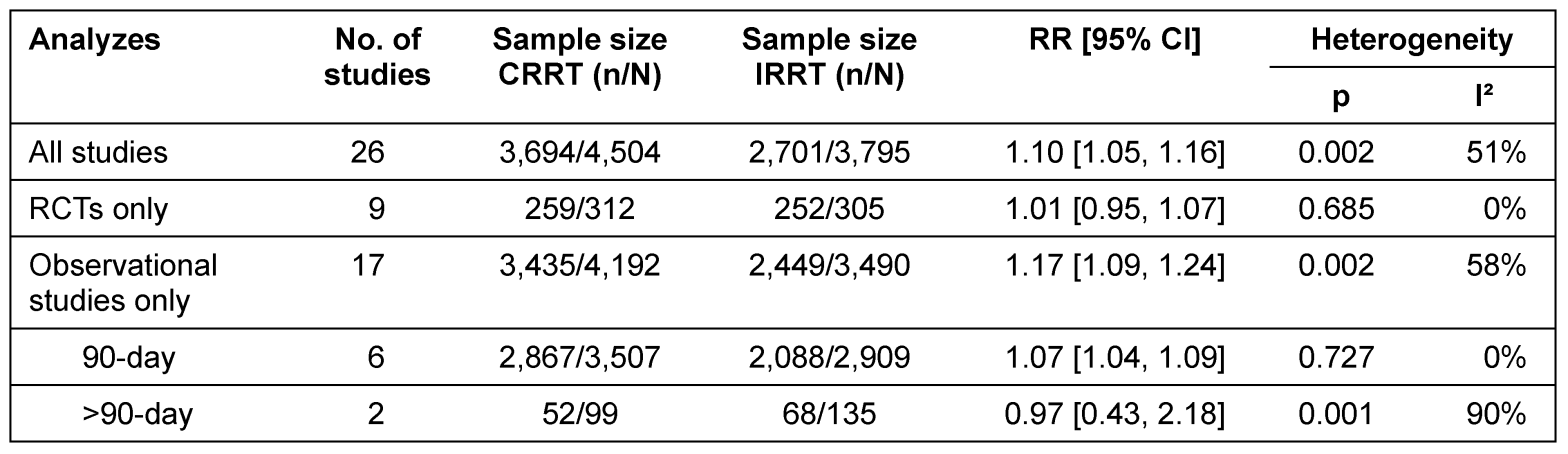
Effect-size summary statistics for renal recovery among AKI survivors

**Table 3:**
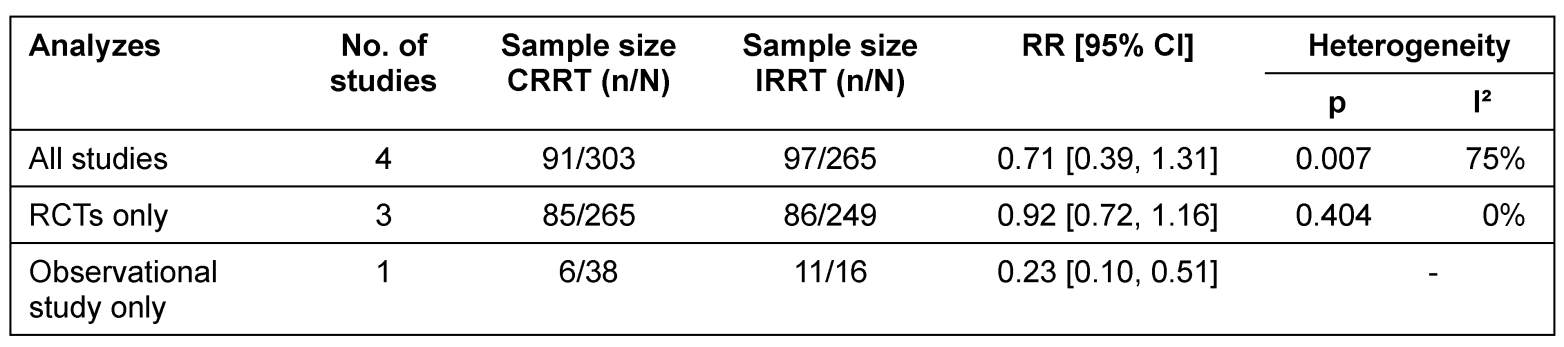
Effect-size summary statistics for hypotension

**Table 4:**
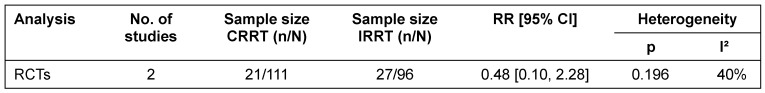
Effect-size summary statistics for hemodynamic instability

**Table 5:**
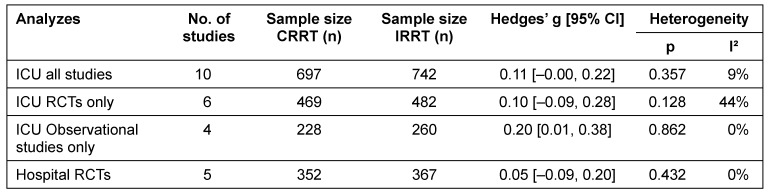
Effect-size summary statistics for length of stay

**Table 6:**
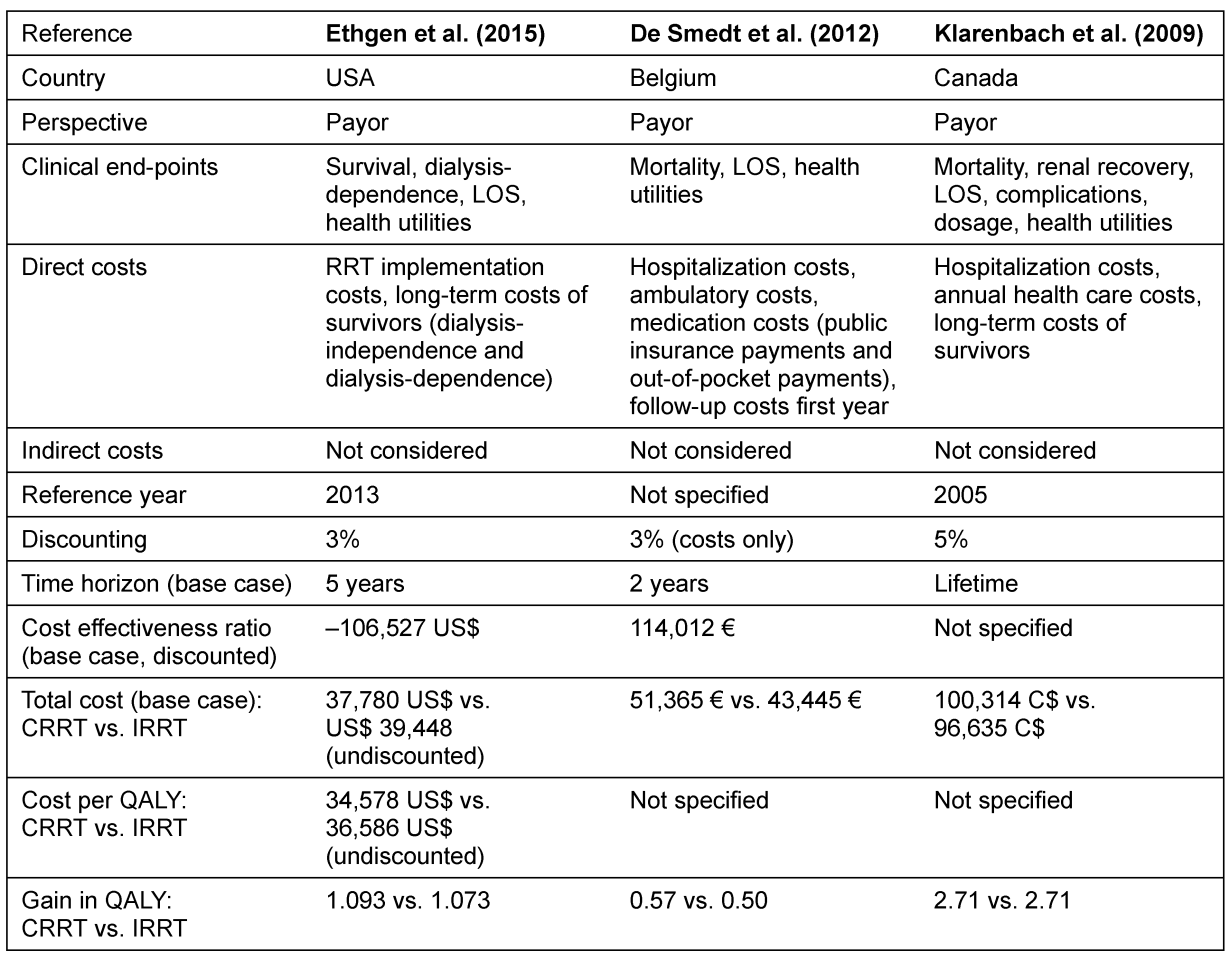
Data on cost-effectiveness studies comparing CRRT and IRRT
