# Supplementary material for: Effects of continuous and intermittent renal replacement therapies among adult patients with acute kidney injury
Source: GMS Health Technol Assess. 2017 Mar 1;13:Doc01. doi: 10.3205/hta000127 (PMC5332811; doi:10.3205/hta000127)
Supplement: Included studies for assessed outcomes [file HTA-13-01-s-001.pdf]

Attachment 1: Included studies for assessed outcomes

Outcome	Included studies
Mortality	
	Abe M, Maruyama N, Matsumoto S, Okada K, Fujita T, Matsumoto K, Soma M. Comparison of sustained hemodiafiltration with acetate-free dialysate and continuous venovenous hemodiafiltration for the treatment of critically ill patients with acute kidney injury. Int J Nephrol 2011;432094.
	Abe M, Okada K, Suzuki M, Nagura C, Ishihara Y, Fujii Y, Ikeda K, Kaizu K, Matsumoto K. Comparison of sustained hemodiafiltration with continuous venovenous hemodiafiltration for the treatment of critically ill patients with acute kidney injury. Artif Organs. 2010;34(4):331-8.
	Augustine JJ, Sandy D, Seifert TH, Paganini EP. A randomized controlled trial comparing intermittent with continuous dialysis in patients with ARF. Am J Kidney. 2004;44(6):1000-7.
	Bagshaw SM, Mortis G, Godinez-Luna T, Doig CJ, Laupland KB. Renal recovery after severe acute renal failure. Int J Artif Organs. 2006;29(11):1023-30.
	Bastien O, Saroul C, Hercule C, George M, Estanove S. Continuous venovenous hemodialysis after cardiac surgery. Contrib Nephrol. 1991;93:76-8.
	Bell M, Granath F, Schon S, Ekbom A, Martling CR. Continuous renal replacement therapy is associated with less chronic renal failure than intermittent haemodialysis after acute renal failure. Intensive Care Med. 2007;33(5):773-80.
	Boulain T, Delpech M, Legras A, Lanotte R, Dequin PF, Perrotin D. Continuous venovenous haemodiafiltration in acute renal failure associated with multiple organ failure: Influence on outcome. Clin Intensive Care. 1996;7(1):4-10.
	Cartin-Ceba R, Haugen EN, Iscimen R, Trillo-Alvarez C, Juncos L, Gajic O. Evaluation of "Loss" and "End stage renal disease" after acute kidney injury defined by the Risk, Injury, Failure, Loss and ESRD classification in critically ill patients. Intensive Care Med. 2009;35(12):2087-95.
	Chang JW, Yang WS, Seo JW, Lee JS, Lee SK, Park SK. Continuous venovenous hemodiafiltration versus hemodialysis as renal replacement therapy in patients with acute renal failure in the intensive care unit. Scand J Urol Nephrol. 2004;38(5):417-21.
	Chen X, Ma T. Sustained low-efficiency daily diafiltration for diabetic nephropathy patients with acute kidney injury. Med Princ Pract. 2014;23(2):119-24.
	Cho KC, Himmelfarb J, Paganini E, Ikizler TA, Soroko SH, Mehta RL, Chertow GM. Survival by dialysis modality in critically ill patients with acute kidney injury. J Am Soc Nephrol. 2006;17(11): 3132-8.
	Delannoy B, Floccard B, Thiolliere F, Kaaki M, Badet M, Rosselli S, Ber CE, Saez A, Flandreau G, Guerin C. Six-month outcome in acute kidney injury requiring renal replacement therapy in the ICU: a multicentre prospective study. Intensive Care Med. 2009;35(11):1907-15.
	Fieghen HE, Friedrich JO, Burns KE, Nisenbaum R, Adhikari NK, Hladunewich MA, Lapinsky SE, Richardson RM, Wald R. The hemodynamic tolerability and feasibility of sustained low efficiency dialysis in the management of critically ill patients with acute kidney injury. BMC Nephrol. 2010;11:32.
	Gangji AS, Rabbat CG, Margetts PJ. Benefit of continuous renal replacement therapy in subgroups of acutely ill patients: a retrospective analysis. Clin Nephrol. 2005;63(4):267-75.
	Gasparovic V, Filipovic-Grcic I, Merkler M, Pisl Z. Continuous renal replacement therapy (CRRT) or intermittent hemodialysis (IHD) - what is the procedure of choice in critically ill patients? Ren Fail. 2003;25(5):855-62.

	Guerin C, Girard R, Selli JM, Ayzac L. Intermittent versus continuous renal replacement therapy for acute renal failure in intensive care units: results from a multicenter prospective epidemiological survey. Intensive Care Med. 2002;28(10):1411-8.
	Hirayama Y, Hirasawa H, Oda S, Shiga H, Nakanishi K, Matsuda K, Nakamura M, Hirano T, Moriguchi T, Watanabe E, Nitta M, Abe R, Nakada T. The change in renal replacement therapy on acute renal failure in a general intensive care unit in a university hospital and its clinica efficacy: a Japanese experience. Ther Apher Dial. 2003;7(5):475-82.
	Jacka MJ, Ivancinova X, Gibney RT. Continuous renal replacement therapy improves renal recovery from acute renal failure. Can J Anaesth. 2005;52(3):327-32.
	John S, Griesbach D, Baumgartel M, Weihprecht H, Schmieder RE, Geiger H. Effects of continuous haemofiltration vs intermittent haemodialysis on systemic haemodynamics and splanchnic regional perfusion in septic shock patients: a prospective, randomized clinical trial. Nephrol Dial Transplant. 2011;16(2):320-7.
	Kumar VA, Craig M, Depner TA, Yeun JY. Extended daily dialysis: A new approach to renal replacement for acute renal failure in the intensive care unit. Am J Kidney Dis. 2000;36(2): 294-300.
	Kumar VA, Yeun JY, Depner TA, Don BR. Extended daily dialysis vs. continuous hemodialysis for ICU patients with acute renal failure: a two-year single center report. Int J Artif Organs. 2004;27(5):371-9.
	Lin YF, Ko WJ, Chu TS, Chen YS, Wu VC, Chen YM, Wu MS, Chen YW, Tsai CW, Shiao CC, Li WY, Hu FC, Tsai PR, Tsai TJ, Wu KD. The 90-day mortality and the subsequent renal recovery in critically ill surgical patients requiring acute renal replacement therapy. Am J Surg. 2009;198(3):325-32.
	Lins RL, Elseviers MM, Van der Niepen P, Hoste E, Malbrain ML, Damas P, Devriendt J. Intermittent versus continuous renal replacement therapy for acute kidney injury patients admitted to the intensive care unit: results of a randomized clinical trial. Nephrol Dial Transplant. 2009;24(2):512-8.
	Manns B, Doig CJ, Lee H, Dean S, Tonelli M, Johnson D, Donaldson C. Cost of acute renal failure requiring dialysis in the intensive care unit: clinical and resource implications of renal recovery. Crit Care Med. 2003;31(2):449-55.
	Mehta RL, McDonald B, Gabbai FB, Pahl M, Pascual MTA, Farkas A, Kaplan RM. A randomized clinical trial of continuous versus intermittent dialysis for acute renal failure. Kidney Int. 2001;60(3):1154-63.
	Neveu H, Kleinknecht D, Brivet F, Loirat P, Landais P. Prognostic factors in acute renal failure due to sepsis. Results of a prospective multicentre study. The French Study Group on Acute Renal Failure. Nephrol Dial Transplant. 1996;11(2):293-9.
	Noble JS, Simpson K, Allison M. Long-term quality of life and hospital mortality in patients treated with intermittent or continuous hemodialysis for acute renal and respiratory failure. Ren Fail. 2006;28(4):323-30.
	Rauf AA, Long KH, Gajic O, Anderson SS, Swaminathan L, Albright RC. Intermittent hemodialysis versus continuous renal replacement therapy for acute renal failure in the intensive care unit: an observational outcomes analysis. J Intensive Care Med. 2008; 23(3):195-203.
	Schefold JC, Haehling S, Pschowski R, Bender T, Berkmann C, Briegel S, Hasper D, Jorres A. The effect of continuous versus intermittent renal replacement therapy on the outcome of critically ill patients with acute renal failure (CONVINT): a prospective randomized controlled trial. Crit Care. 2014;18(1):R11.

	Schwenger V, Weigand MA, Hoffmann O, Dikow R, Kihm LP, Seckinger J, Miftari N, Schaier M, Hofer S, Haar C, Nawroth PP, Zeier M, Martin E, Morath C. Sustained low efficiency dialysis using a single-pass batch system in acute kidney injury - a randomized interventional trial: the REnal Replacement Therapy Study in Intensive Care Unit PatiEnts. Crit Care. 2012;16(4):R140.
	Sun Z, Ye H, Shen X, Chao H, Wu X, Yang J. Continuous venovenous hemofiltration versus extended daily hemofiltration in patients with septic acute kidney injury: a retrospective cohort study. Crit Care. 2014;18(2):R70.
	Swartz RD, Bustami RT, Daley JM, Gillespie BW, Port FK. Estimating the impact of renal replacement therapy choice on outcome in severe acute renal failure. Clin Nephrol. 2005;63(5):335-45.
	Swartz RD, Messana JM, Orzol S, Port FK. Comparing continuous hemofiltration with hemodialysis in patients with severe acute renal failure. Am J Kidney Dis. 1999;34(3):424-32.
	Uchino S, Bellomo R, Kellum JA, Morimatsu H, Morgera S, Schetz MR, Tan I, Bouman C, Macedo E, Gibney N, Tolwani A, Oudemans-Van Straaten HM, Ronco C. Patient and kidney survival by dialysis modality in critically ill patients with acute kidney injury. Int J Artif Organs. 2007;30(4):281-92.
	Uehlinger DE, Jakob SM, Ferrari P, Eichelberger M, Huynh-Do U, Marti HP, Mohaupt MG, Vogt B, Rothen HU, Regli B, Takala J, Frey FJ. Comparison of continuous and intermittent renal replacement therapy for acute renal failure. Nephrol Dial Transplant. 2005;20(8):1630-7.
	Vinsonneau C, Camus C, Combes A, Costa de Beauregard MA, Klouche K, Boulain T, Pallot JL, Chiche JD, Taupin P, Landais P, Dhainaut JF. Continuous venovenous haemodiafiltration versus intermittent haemodialysis for acute renal failure in patients with multiple-organ dysfunction syndrome: a multicentre randomised trial. Lancet. 2006;368(9533):379-85.
	Wald R, Shariff SZ, Adhikari NK, Bagshaw SM, Burns KE, Friedrich JO, Garg AX, Harel Z, Kitchlu A, Ray JG. The association between renal replacement therapy modality and long-term outcomes among critically ill adults with acute kidney injury: a retrospective cohort study. Crit Care Med. 2014;42(4):868-77.
	Waldrop J, Ciraulo DL, Milner TP, Gregori D, Kendrick AS, Richart CM, Maxwell RA, Barker DE. A comparison of continuous renal replacement therapy to intermittent dialysis in the management of renal insufficiency in the acutely III surgical patient. Am Surg. 2005;71(1):36-9.
	Wu HHL, Chan KKC, Lau ACW, Yan WW. Outcomes of acute renal failure patients having received renal replacement therapy in the Intensive Care Unit. Crit Care & Shock. 2010;13:81-90.
	Wu VC, Shiao CC, Chang CH, Huang TM, Lai CF, Lin MC, Chiang WC, Chu TS, Wu KD, Ko WJ, Wang CY, Wang SM, Chen L. Long-term outcomes after dialysis-requiring acute kidney injury. Biomed Res Int. 2014;365186.
Renal Recovery	
	Abe M, Maruyama N, Matsumoto S, Okada K, Fujita T, Matsumoto K, Soma M. Comparison of sustained hemodiafiltration with acetate-free dialysate and continuous venovenous hemodiafiltration for the treatment of critically ill patients with acute kidney injury. Int J Nephrol 2011;432094.
	Abe M, Okada K, Suzuki M, Nagura C, Ishihara Y, Fujii Y, Ikeda K, Kaizu K, Matsumoto K. Comparison of sustained hemodiafiltration with continuous venovenous hemodiafiltration for the treatment of critically ill patients with acute kidney injury. Artif Organs. 2010;34(4):331-8.
	Augustine JJ, Sandy D, Seifert TH, Paganini EP. A randomized controlled trial comparing intermittent with continuous dialysis in patients with ARF. Am J Kidney. 2004;44(6):1000-7.

	Bagshaw SM, Mortis G, Godinez-Luna T, Doig CJ, Laupland KB. Renal recovery after severe acute renal failure. Int J Artif Organs. 2006;29(11):1023-30.
	Bell M, Granath F, Schon S, Ekbom A, Martling CR. Continuous renal replacement therapy is associated with less chronic renal failure than intermittent haemodialysis after acute renal failure. Intensive Care Med. 2007;33(5):773-80.
	Boulain T, Delpech M, Legras A, Lanotte R, Dequin PF, Perrotin D. Continuous venovenous haemodiafiltration in acute renal failure associated with multiple organ failure: Influence on outcome. Clin Intensive Care. 1996;7(1):4-10.
	Cartin-Ceba R, Haugen EN, Iscimen R, Trillo-Alvarez C, Juncos L, Gajic O. Evaluation of "Loss" and "End stage renal disease" after acute kidney injury defined by the Risk, Injury, Failure, Loss and ESRD classification in critically ill patients. Intensive Care Med. 2009;35(12):2087-95.
	Chang JW, Yang WS, Seo JW, Lee JS, Lee SK, Park SK. Continuous venovenous hemodiafiltration versus hemodialysis as renal replacement therapy in patients with acute renal failure in the intensive care unit. Scand J Urol Nephrol. 2004;38(5):417-21.
	Delannoy B, Floccard B, Thiolliere F, Kaaki M, Badet M, Rosselli S, Ber CE, Saez A, Flandreau G, Guerin C. Six-month outcome in acute kidney injury requiring renal replacement therapy in the ICU: a multicentre prospective study. Intensive Care Med. 2009;35(11):1907-15.
	Gangji AS, Rabbat CG, Margetts PJ. Benefit of continuous renal replacement therapy in subgroups of acutely ill patients: a retrospective analysis. Clin Nephrol. 2005;63(4):267-75.
	Jacka MJ, Ivancinova X, Gibney RT. Continuous renal replacement therapy improves renal recovery from acute renal failure. Can J Anaesth. 2005;52(3):327-32.
	Kumar VA, Yeun JY, Depner TA, Don BR. Extended daily dialysis vs. continuous hemodialysis for ICU patients with acute renal failure: a two-year single center report. Int J Artif Organs. 2004;27(5):371-9.
	Lin YF, Ko WJ, Chu TS, Chen YS, Wu VC, Chen YM, Wu MS, Chen YW, Tsai CW, Shiao CC, Li WY, Hu FC, Tsai PR, Tsai TJ, Wu KD. The 90-day mortality and the subsequent renal recovery in critically ill surgical patients requiring acute renal replacement therapy. Am J Surg. 2009;198(3):325-32.
	Lins RL, Elseviers MM, Van der Niepen P, Hoste E, Malbrain ML, Damas P, Devriendt J. Intermittent versus continuous renal replacement therapy for acute kidney injury patients admitted to the intensive care unit: results of a randomized clinical trial. Nephrol Dial Transplant. 2009;24(2):512-8.
	Manns B, Doig CJ, Lee H, Dean S, Tonelli M, Johnson D, Donaldson C. Cost of acute renal failure requiring dialysis in the intensive care unit: clinical and resource implications of renal recovery. Crit Care Med. 2003;31(2):449-55.
	Mehta RL, McDonald B, Gabbai FB, Pahl M, Pascual MTA, Farkas A, Kaplan RM. A randomized clinical trial of continuous versus intermittent dialysis for acute renal failure. Kidney Int. 2001;60(3):1154-63.
	Rauf AA, Long KH, Gajic O, Anderson SS, Swaminathan L, Albright RC. Intermittent hemodialysis versus continuous renal replacement therapy for acute renal failure in the intensive care unit: an observational outcomes analysis. J Intensive Care Med. 2008; 23(3):195-203.
	Schefold JC, Haehling S, Pschowski R, Bender T, Berkmann C, Briegel S, Hasper D, Jorres A. The effect of continuous versus intermittent renal replacement therapy on the outcome of critically ill patients with acute renal failure (CONVINT): a prospective randomized controlled trial. Crit Care. 2014;18(1):R11.

	Sun Z, Ye H, Shen X, Chao H, Wu X, Yang J. Continuous venovenous hemofiltration versus extended daily hemofiltration in patients with septic acute kidney injury: a retrospective cohort study. Crit Care. 2014;18(2):R70.
	Swartz RD, Bustami RT, Daley JM, Gillespie BW, Port FK. Estimating the impact of renal replacement therapy choice on outcome in severe acute renal failure. Clin Nephrol. 2005;63(5):335-45.
	Swartz RD, Messana JM, Orzol S, Port FK. Comparing continuous hemofiltration with hemodialysis in patients with severe acute renal failure. Am J Kidney Dis. 1999;34(3):424-32.
	Uchino S, Bellomo R, Kellum JA, Morimatsu H, Morgera S, Schetz MR, Tan I, Bouman C, Macedo E, Gibney N, Tolwani A, Oudemans-Van Straaten HM, Ronco C. Patient and kidney survival by dialysis modality in critically ill patients with acute kidney injury. Int J Artif Organs. 2007;30(4):281-92.
	Uehlinger DE, Jakob SM, Ferrari P, Eichelberger M, Huynh-Do U, Marti HP, Mohaupt MG, Vogt B, Rothen HU, Regli B, Takala J, Frey FJ. Comparison of continuous and intermittent renal replacement therapy for acute renal failure. Nephrol Dial Transplant. 2005;20(8):1630-7.
	Vinsonneau C, Camus C, Combes A, Costa de Beauregard MA, Klouche K, Boulain T, Pallot JL, Chiche JD, Taupin P, Landais P, Dhainaut JF. Continuous venovenous haemodiafiltration versus intermittent haemodialysis for acute renal failure in patients with multiple-organ dysfunction syndrome: a multicentre randomised trial. Lancet. 2006;368(9533):379-85.
	Wald R, Shariff SZ, Adhikari NK, Bagshaw SM, Burns KE, Friedrich JO, Garg AX, Harel Z, Kitchlu A, Ray JG. The association between renal replacement therapy modality and long-term outcomes among critically ill adults with acute kidney injury: a retrospective cohort study. Crit Care Med. 2014;42(4):868-77.
	Waldrop J, Ciraulo DL, Milner TP, Gregori D, Kendrick AS, Richart CM, Maxwell RA, Barker DE. A comparison of continuous renal replacement therapy to intermittent dialysis in the management of renal insufficiency in the acutely III surgical patient. Am Surg. 2005;71(1):36-9.
Change in mean arterial pressure	
	Augustine JJ, Sandy D, Seifert TH, Paganini EP. A randomized controlled trial comparing intermittent with continuous dialysis in patients with ARF. Am J Kidney. 2004;44(6):1000-7.
Hypotension	
	John S, Griesbach D, Baumgartel M, Weihprecht H, Schmieder RE, Geiger H. Effects of continuous haemofiltration vs intermittent haemodialysis on systemic haemodynamics and splanchnic regional perfusion in septic shock patients: a prospective, randomized clinical trial. Nephrol Dial Transplant. 2011;16(2):320-7.
	Hirayama Y, Hirasawa H, Oda S, Shiga H, Nakanishi K, Matsuda K, Nakamura M, Hirano T, Moriguchi T, Watanabe E, Nitta M, Abe R, Nakada T. The change in renal replacement therapy on acute renal failure in a general intensive care unit in a university hospital and its clinica efficacy: a Japanese experience. Ther Apher Dial. 2003;7(5):475-82.
	Uehlinger DE, Jakob SM, Ferrari P, Eichelberger M, Huynh-Do U, Marti HP, Mohaupt MG, Vogt B, Rothen HU, Regli B, Takala J, Frey FJ. Comparison of continuous and intermittent renal replacement therapy for acute renal failure. Nephrol Dial Transplant. 2005;20(8):1630-7.
	Vinsonneau C, Camus C, Combes A, Costa de Beauregard MA, Klouche K, Boulain T, Pallot JL, Chiche JD, Taupin P, Landais P, Dhainaut JF. Continuous venovenous haemodiafiltration versus intermittent haemodialysis for acute renal failure in patients with multiple-organ dysfunction syndrome: a multicentre randomised trial. Lancet. 2006;368(9533):379-85.

Hemodynamic instability	
	Augustine JJ, Sandy D, Seifert TH, Paganini EP. A randomized controlled trial comparing intermittent with continuous dialysis in patients with ARF. Am J Kidney. 2004;44(6):1000-7.
	Uehlinger DE, Jakob SM, Ferrari P, Eichelberger M, Huynh-Do U, Marti HP, Mohaupt MG, Vogt B, Rothen HU, Regli B, Takala J, Frey FJ. Comparison of continuous and intermittent renal replacement therapy for acute renal failure. Nephrol Dial Transplant. 2005;20(8):1630-7.
Fluid balance	
	Augustine JJ, Sandy D, Seifert TH, Paganini EP. A randomized controlled trial comparing intermittent with continuous dialysis in patients with ARF. Am J Kidney. 2004;44(6):1000-7.
	John S, Griesbach D, Baumgartel M, Weihprecht H, Schmieder RE, Geiger H. Effects of continuous haemofiltration vs intermittent haemodialysis on systemic haemodynamics and splanchnic regional perfusion in septic shock patients: a prospective, randomized clinical trial. Nephrol Dial Transplant. 2011;16(2):320-7.
	Uehlinger DE, Jakob SM, Ferrari P, Eichelberger M, Huynh-Do U, Marti HP, Mohaupt MG, Vogt B, Rothen HU, Regli B, Takala J, Frey FJ. Comparison of continuous and intermittent renal replacement therapy for acute renal failure. Nephrol Dial Transplant. 2005;20(8):1630-7.
	Schefold JC, Haehling S, Pschowski R, Bender T, Berkmann C, Briegel S, Hasper D, Jorres A. The effect of continuous versus intermittent renal replacement therapy on the outcome of critically ill patients with acute renal failure (CONVINT): a prospective randomized controlled trial. Crit Care. 2014;18(1):R11.
Length of stay	
	Abe M, Maruyama N, Matsumoto S, Okada K, Fujita T, Matsumoto K, Soma M. Comparison of sustained hemodiafiltration with acetate-free dialysate and continuous venovenous hemodiafiltration for the treatment of critically ill patients with acute kidney injury. Int J Nephrol 2011;432094.
	Abe M, Okada K, Suzuki M, Nagura C, Ishihara Y, Fujii Y, Ikeda K, Kaizu K, Matsumoto K. Comparison of sustained hemodiafiltration with continuous venovenous hemodiafiltration for the treatment of critically ill patients with acute kidney injury. Artif Organs. 2010;34(4):331-8.
	Boulain, T., Delpech, M., Legras, A., Lanotte, R., Dequin, P. F. und Perrotin, D. Continuous venovenous haemodiafiltration in acute renal failure associated with multiple organ failure: Influence on outcome. Clin Intensive Care. 1996;7(1):4-10.
	Khanal N, Marshall MR, Ma TM, Pridmore PJ, Williams AB, Rankin AP. Comparison of outcomes by modality for critically ill patients requiring renal replacement therapy: a single-centre cohort study adjusting for time-varying illness severity and modality exposure. Anaesth Intensive Care; 2012;40(2):260-8.
	Mehta RL, McDonald B, Gabbai FB, Pahl M, Pascual MTA, Farkas A, Kaplan RM. A randomized clinical trial of continuous versus intermittent dialysis for acute renal failure. Kidney Int. 2001;60(3):1154-63.
	Rauf AA, Long KH, Gajic O, Anderson SS, Swaminathan L, Albright RC. Intermittent hemodialysis versus continuous renal replacement therapy for acute renal failure in the intensive care unit: an observational outcomes analysis. J Intensive Care Med. 2008; 23(3):195-203.
	Schefold JC, Haehling S, Pschowski R, Bender T, Berkmann C, Briegel S, Hasper D, Jorres A. The effect of continuous versus intermittent renal replacement therapy on the outcome of critically ill patients with acute renal failure (CONVINT): a prospective randomized controlled trial. Crit Care. 2014;18(1):R11.

	Schwenger V, Weigand MA, Hoffmann O, Dikow R, Kihm LP, Seckinger J, Miftari N, Schaier M, Hofer S, Haar C, Nawroth PP, Zeier M, Martin E, Morath C. Sustained low efficiency dialysis using a single-pass batch system in acute kidney injury - a randomized interventional trial: the REnal Replacement Therapy Study in Intensive Care Unit PatiEnts. Crit Care. 2012;16(4):R140.
	Vinsonneau C, Camus C, Combes A, Costa de Beauregard MA, Klouche K, Boulain T, Pallot JL, Chiche JD, Taupin P, Landais P, Dhainaut JF. Continuous venovenous haemodiafiltration versus intermittent haemodialysis for acute renal failure in patients with multiple-organ dysfunction syndrome: a multicentre randomised trial. Lancet. 2006;368(9533):379-85.
Cost-effectiveness	
	Klarenbach S, Manns B, Pannu N, Clement FM, Wiebe N, Tonelli M. Economic evaluation of continuous renal replacement therapy in acute renal failure. Int J Technol Assess Health Care. 2009;25(3):331-8.
	De Smedt DM, Elseviers MM, Lins RL, Annemans L. Economic evaluation of different treatment modalities in acute kidney injury. Nephrol Dial Transplant; 2012;27(11):4095-101.
	Ethgen O, Schneider AG, Bagshaw SM, Bellomo R, Kellum JA. Economics of dialysis dependence following renal replacement therapy for critically ill acute kidney injury patients. Nephrol Dial Transplant. 2015;30(1):54-61.
Ethical and social aspects	
	Patel SS, Holley JL. Withholding and withdrawing dialysis in the intensive care unit: benefits derived from consulting the renal physicians association/american society of nephrology clinical practice guideline, shared decision-making in the appropriate initiation of and withdrawal from dialysis. Clin J Am Soc Nephrol. 2008;3(2):587-93.
	Wenger NS, Lynn J, Oye RK, Liu H, Teno JM, Phillips RS, Desbiens NA, Sehgal A, Kussin P, Taub H, Harrell F, Knaus W. Withholding versus withdrawing life-sustaining treatment: patient factors and documentation associated with dialysis decisions. J Am Geriatr Soc. 2000;48(5 Suppl):S75-83.
	Legrand M, Darmon M, Joannidis M, Payen D. Management of renal replacement therapy in ICU patients: an international survey. Intensive Care Med. 2013;39(1):101-8.
	Schindler R, Hutagalung R, Jorres A, John S, Quintel MI, Brunkhorst FM, Heering P. Treatment of acute renal failure in Germany: a structural analysis. Dtsch Med Wochenschr. 2014;139(34-35):1701-6.
